# Left Brain, Right Brain: Facts and Fantasies

**DOI:** 10.1371/journal.pbio.1001767

**Published:** 2014-01-21

**Authors:** Michael C. Corballis

**Affiliations:** School of Psychology, University of Auckland, Auckland, New Zealand

## Abstract

Handedness and brain asymmetry are widely regarded as unique to humans, and associated with complementary functions such as a left-brain specialization for language and logic and a right-brain specialization for creativity and intuition. In fact, asymmetries are widespread among animals, and support the gradual evolution of asymmetrical functions such as language and tool use. Handedness and brain asymmetry are inborn and under partial genetic control, although the gene or genes responsible are not well established. Cognitive and emotional difficulties are sometimes associated with departures from the “norm” of right-handedness and left-brain language dominance, more often with the absence of these asymmetries than their reversal.

## Abstract 2

Michael Corballis discusses in this essay how the asymmetry of the brain raises questions about genetics, evolution, language, and educational and psychological disabilities; but beware of exaggerated claims of left brain/right brain polarities.

*“That raven on yon left-hand oak*

*(Curse his ill-betiding croak)*

*Bodes me no good!”*

*—from* Fables, *by John Gay (1688–1732)*

## Introduction

The most obvious sign that our brains function asymmetrically is the near-universal preference for the right hand, which goes back at least as far as the historical record takes us, and has long been a powerful source of symbolism, with the dexterous right associated with positive values and the sinister left with negative ones [1]. This has often led to stigmatization of left-handed individuals, sometimes forcing them to switch hand use, occasionally with grievous consequences. Superstitions about left and right were compounded by the discovery, in the 1860s, that speech was based predominantly in the left hemisphere of the brain [2]. Since language itself is uniquely human, this reinforced the idea that brain asymmetry more generally is a distinctive mark of being human [3]. Because the left hemisphere also controls the dominant right hand, it came to be widely regarded as the dominant or major hemisphere, and the right as nondominant or minor. Nevertheless, further evidence that the right hemisphere was the more specialized for perception and emotion also led to speculation, some of it far-fetched, about the complementary roles of the two sides of the brain in maintaining psychological equilibrium [4].

Interest flagged for a while, but was revived a century later, in the 1960s, with the study of patients who had undergone split-brain surgery, in which the main commissures connecting the two hemispheres were cut as a means of controlling intractable epilepsy. Testing of each disconnected hemisphere again revealed the left to be specialized for language and the right for emotional and nonverbal functions [5],[6]. This work won Roger W. Sperry the Nobel Prize for Physiology and Medicine in 1981, but again led to speculation, most of it exaggerated or ill-founded, about the complementary functions of the two sides of the brain.

One popular example is Betty Edwards' *Drawing on the Right Side of the Brain*, first published in 1979 but now in its fourth edition [7], which epitomizes the popular view that the right hemisphere is responsible for creativity. Brain imaging shows, though, that creative thought activates a widespread network, favoring neither hemisphere [8]. A more recent example is Iain McGilchrist's 2009 book *The Master and His Emissary*, which draws on cerebral asymmetry in a sweeping account of the forces that shaped Western culture, and provocatively declares the right hemisphere to be the dominant one (“the master”) [9]. Although widely acclaimed, this book goes far beyond the neurological facts. Polarities of left and right brain are broadly invoked in art, business, education, literary theory, and culture, but owe more to the power of myth than to the scientific evidence [10].

## Evolution of Brain Asymmetries, with Implications for Language

**Figure 1:**
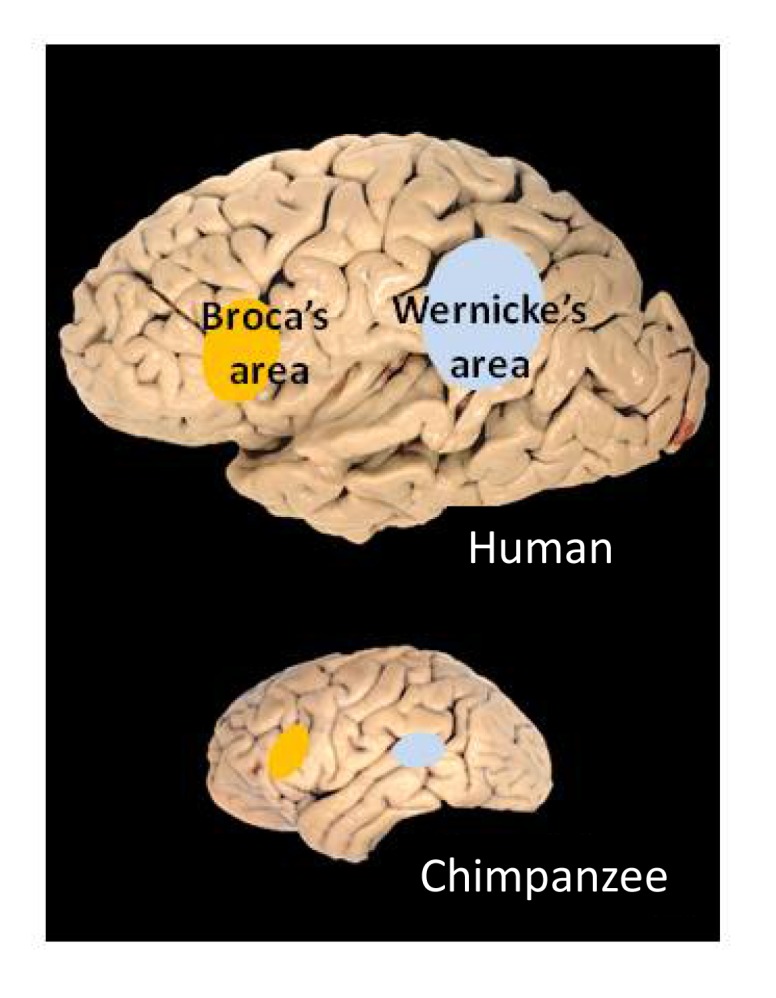
Human brain showing Broca's and Wernicke's areas (upper diagram) and areas of chimpanzee brain showing leftward enlargement (lower diagram). Image credit: Todd Preuss, Yerkes Primate Research Center (http://commons.wikimedia.org/wiki/File:Human_and_chimp_brain.png).

One myth that persists even in some scientific circles is that asymmetry is uniquely human [3]. Left–right asymmetries of brain and behavior are now known to be widespread among both vertebrates and invertebrates [11], and can arise through a number of genetic, epigenetic, or neural mechanisms [12]. Many of these asymmetries parallel those in humans, or can be seen as evolutionary precursors. A strong left-hemispheric bias for action dynamics in marine mammals and in some primates and the left-hemisphere action biases in humans, perhaps including gesture, speech, and tool use, may derive from a common precursor [13]. A right-hemisphere dominance for emotion seems to be present in all primates so far investigated, suggesting an evolutionary continuity going back at least 30 to 40 million years [14]. A left-hemisphere dominance for vocalization has been shown in mice [15] and frogs [16], and may well relate to the leftward dominance for speech—although language itself is unique to humans and is not necessarily vocal, as sign languages remind us. Around two-thirds of chimpanzees are right-handed, especially in gesturing [17] and throwing [18], and also show left-sided enlargement in two cortical areas homologous to the main language areas in humans—namely, Broca's area [19] and Wernicke's area [20] (see Figure 1). These observations have been taken as evidence that language did not appear de novo in humans, as argued by Chomsky [21] and others, but evolved gradually through our primate lineage [22]. They have also been interpreted as evidence that language evolved not from primate calls, but from manual gestures [23]–[25].

Some accounts of language evolution (e.g., [25]) have focused on mirror neurons, first identified in the monkey brain in area F5 [26], a region homologous to Broca's area in humans, but now considered part of an extensive network more widely homologous to the language network [27]. Mirror neurons are so called because they respond when the monkey performs an action, and also when they see another individual performing the same action. This “mirroring” of what the monkey sees onto what it does seems to provide a natural platform for the evolution of language, which likewise can be seen to involve a mapping of perception onto production. The motor theory of speech perception, for example, holds that we perceive speech sounds according to how we produce them, rather than through acoustic analysis [28]. Mirror neurons in monkeys also respond to the sounds of such physical actions as ripping paper or dropping a stick onto the floor, but they remain silent to animal calls [29]. This suggests an evolutionary trajectory in which mirror neurons emerged as a system for producing and understanding manual actions, but in the course of evolution became increasingly lateralized to the left brain, incorporating vocalization and gaining grammar-like complexity [30]. The left hemisphere is dominant for sign language as for spoken language [31].

Mirror neurons themselves have been victims of hyperbole and myth [32], with the neuroscientist Vilayanur Ramachandran once predicting that “mirror neurons will do for psychology what DNA did for biology” [33]. As the very name suggests, mirror neurons are often taken to be the basis of imitation, yet nonhuman primates are poor imitators. Further, the motor theory of speech perception does not account for the fact that speech can be understood by those deprived of the ability to speak, such as those with damage to Broca's area. Even chimpanzees [34] and dogs [35] can learn to respond to simple spoken instructions, but cannot produce anything resembling human speech. An alternative is that mirror neurons are part of a system for calibrating movements to conform to perception, as a process of learning rather than direct imitation. A monkey repeatedly observes its hand movements to learn to reach accurately, and the babbling infant calibrates the production of sounds to match what she hears. Babies raised in households where sign language is used “babble” by making repetitive movements of the hands [36]. Moreover, it is this productive aspect of language, rather than the mechanisms of understanding, that shows the more pronounced bias to the left hemisphere [37].

## Inborn Asymmetries

**Figure 2:**
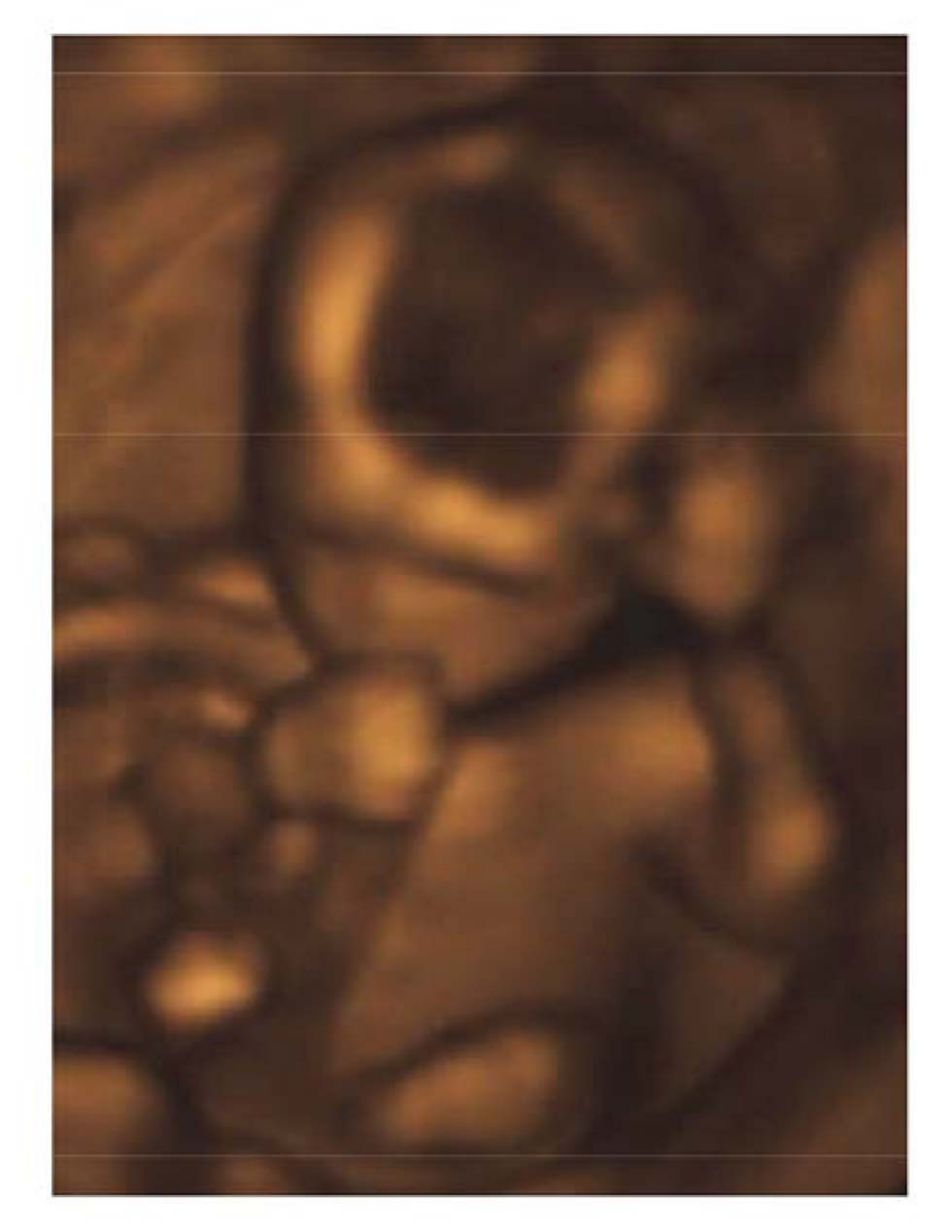
Ultrasound image of a fetus sucking the right thumb. Image credit: jenny cu (http://commons.wikimedia.org/wiki/File:Sucking_his_thumb_and_waving.jpg).

Handedness and cerebral asymmetries are detectable in the fetus. Ultrasound recording has shown that by the tenth week of gestation, the majority of fetuses move the right arm more than the left [38], and from the 15th week most suck the right thumb rather than the left [39]—an asymmetry strongly predictive of later handedness [40] (see Figure 2). In the first trimester, a majority of fetuses show a leftward enlargement of the choroid plexus [41], a structure within the ventricles known to synthesize peptides, growth factors, and cytokines that play a role in neurocortical development [42]. This asymmetry may be related to the leftward enlargement of the temporal planum (part of Wernicke's area), evident at 31 weeks [43].

In these prenatal brain asymmetries, around two-thirds of cases show the leftward bias. The same ratio applies to the asymmetry of the temporal planum in both infants and adults [44]. The incidence of right-handedness in the chimpanzee is also around 65–70 percent, as is a clockwise torque, in which the right hemisphere protrudes forwards and the left hemisphere rearwards, in both humans and great apes [45]. These and other asymmetries have led to the suggestion that a “default” asymmetry of around 65–70 percent, in great apes as well as humans, is inborn, with the asymmetry of human handedness and cerebral asymmetry for language increased to around 90 percent by “cultural literacy” [46].

## Variations in Asymmetry

Whatever their “true” incidence, variations in handedness and cerebral asymmetry raise doubts as to the significance of the “standard” condition of right-handedness and left-cerebral specialization for language, along with other qualities associated with the left and right brains that so often feature in popular discourse. Handedness and cerebral asymmetry are not only variable, they are also imperfectly related. Some 95–99 percent of right-handed individuals are left-brained for language, but so are about 70 percent of left-handed individuals. Brain asymmetry for language may actually correlate more highly with brain asymmetry for skilled manual action, such as using tools [47],[48], which again supports the idea that language itself grew out of manual skill—perhaps initially through pantomime.

Even when the brain is at rest, brain imaging shows that there are asymmetries of activity in a number of regions. A factor analysis of these asymmetries revealed four different dimensions, each mutually uncorrelated. Only one of these dimensions corresponded to the language regions of the brain; the other three had to do with vision, internal thought, and attention [49]—vision and attention were biased toward the right hemisphere, language and internal thought to the left. This multidimensional aspect throws further doubt on the idea that cerebral asymmetry has some unitary and universal import.

Handedness, at least, is partly influenced by parental handedness, suggesting a genetic component [50], but genes can't tell the whole story. For instance some 23 percent of monozygotic twins, who share the same genes, are of opposite handedness [51]. These so-called “mirror twins” have themselves fallen prey to a *Through the Looking Glass* myth; according to Martin Gardner [52], Lewis Carroll intended the twins Tweedledum and Tweedledee in that book to be enantiomers, or perfect three-dimensional mirror images in bodily form as well as in hand and brain function. Although some have argued that mirroring arises in the process of twinning itself [53],[54], large-scale studies suggest that handedness [55],[56] and cerebral asymmetry [57] in mirror twins are not subject to special mirroring effects. In the majority of twins of opposite handedness the left hemisphere is dominant for language in both twins, consistent with the finding that the majority of single-born left-handed individuals are also left-hemisphere dominant for language. In twins, as in the singly born, it is estimated that only about a quarter of the variation in handedness is due to genetic influences [56].

The manner in which handedness is inherited has been most successfully modeled by supposing that a gene or genes influence not whether the individual is right- or left-handed, but whether a bias to right-handedness will be expressed or not. In those lacking the “right shift” bias, the direction of handedness is a matter of chance; that is, left-handedness arises from the lack of a bias toward the right hand, and not from a “left-hand gene.” Such models can account reasonably well for the parental influence [58]–[60], and even for the relation between handedness and cerebral asymmetry if it is supposed that the same gene or genes bias the brain toward a left-sided dominance for speech [60],[61]. It now seems likely that a number of such genes are involved, but the basic insight that genes influence whether or not a given directional bias is expressed, rather than whether or not it can be reversed, remains plausible (see Box 1).

**Box 1. The Genetics of Handedness and Cerebral Asymmetry**

Linkage analyses have often revealed candidate laterality genes, but all too often these fail in follow-up analysis—a common problem in the search for genes related to human behavior. Part of the problem is the sheer immensity of the genome, which means that candidates are likely to surface by chance, and the problem is compounded by the likelihood of a strong chance element in the determination of handedness itself. With appropriate statistical control, several large-scale genome-wide studies have failed to reveal any single locus to be significantly associated with handedness [68],[69], including one study [70] based on a large sample of twins, which also failed specifically to support the single-gene model developed by McManus [60], or weaker versions of that model. The authors of one study estimate that as many as 40 different loci may be involved [71], but note that it would be difficult to distinguish multilocus models from a single-gene model, such as that of McManus, in terms of handedness pedigrees.

The study of one candidate gene, *PCSK6*, has led to some insight as to polygenic control of handedness. Across three independent samples of individuals with dyslexia, a genome-wide assay revealed the minor allele at the rs11855415 locus within this gene to be significantly associated with increased right-handedness [72]. This allele was not significantly associated with handedness in a large sample from the general population. Another targeted search within the *PCSK6* gene failed to confirm a role for rs11855415 in a large sample from the general population, but revealed that a tandem repeat polymorphism at another locus, rs10523972, was associated with the degree, but not the direction, of handedness [73]. PCSK6 is involved in regulating NODAL, which plays a role in the development of the left–right axis in vertebrates, and knock-out of *PCSK6* in mice results in defects in the placement of normally asymmetrical internal organs. Several other genes in the pathway that leads to anomalies of left–right development in mice proved to be associated as a group with human handedness in the general population, leading to the suggestion that handedness is indeed a polygenic trait partly controlled by the genes that establish body asymmetry early in development [74].

Another gene of interest is *LRRTM1*, which has been associated with handedness and schizophrenia when inherited through the father [75], where a particular haplotype consisting of minor alleles at three locations within the gene significantly shifted handedness to the left—a finding partially confirmed elsewhere [76]. Again, though, *LRRTM1* does not stand out in genome-wide assays in samples from the general population. Nevertheless, schizophrenia has long been associated with increased left-handedness or ambidexterity [77],[78], as have schizotypy and tendencies to magical thinking [79]–[81]. Just as the association of *PCSK6* with dyslexia led to suggestion of a polygenic pathway, so the association of *LRRTM1* with schizophrenia may lead to other pathways influencing handedness and brain asymmetry.

Another suggestion is that cerebral asymmetry, and even a disposition to schizophrenia, was critical to human speciation, involving a rearrangement within the X and Y chromosomes, and that it was this event that constituted the supposed “big bang” that created language de novo in our species [82]. The idea that language emerged in this saltatory fashion, still championed by Chomsky [21], is now widely questioned [83],[84]. Linkage analysis gives little support to the involvement of the X and Y chromosomes, although one study has shown that repeats of a CAG sequence in the androgen receptor locus on the X chromosome are linked to handedness. In females the incidence of left-handedness increased with the number of repeats, while in males it was reduced with the number of repeats. This finding supports a role for testosterone in the determination of handedness [85]. In recent formulations of the X–Y theory, it has been proposed that handedness and cerebral asymmetry are facultative traits, universally encoded in the human genome, and that the variations giving rise to schizophrenia or anomalies of handedness and cerebral asymmetry are epigenetic, and therefore not coded in the nucleotide sequence [86]. It appears that epigenetic change through DNA methylation can be transmitted between generations [87], which might explain pedigree effects that are not detected in linkage analyses.

Another gene that has been linked to language evolution is the *FOXP2* gene, following the discovery that about half the members of an extended family possessed a mutation of this gene that caused a severe deficit in articulating speech [88]. Unlike the unaffected family members, they all failed to show activation of Broca's area when asked to silently generate words, and indeed showed no consistent asymmetry at all [89]. A more recent study also shows widespread anatomical differences between the affected and unaffected family members, including bilateral reduction of the caudate nucleus in the affected members, along with a reduction of grey matter in Broca's area on the left [90]. All of the affected individuals are right-handed, though, so the effect of the mutation appears to involve the brain circuits involved in speech, and possibly more generally in language and other motor skills, but not in handedness itself. Although highly conserved in mammalian evolution, the human *FOXP2* gene differs in two locations from that in the chimpanzee, leading to the suggestion that it may have played a role in the evolution of language [91]. Evidence that the most recent mutation was also present in Neanderthal DNA [92] again argues against the “big bang” theory that language evolved uniquely in humans.

Genetic considerations aside, departures from right-handedness or left-cerebral dominance have sometimes been linked to disabilities. In the 1920s and 1930s, the American physician Samuel Torrey Orton attributed both reading disability and stuttering to a failure to establish cerebral dominance [62]. Orton's views declined in influence, perhaps in part because he held eccentric ideas about interhemispheric reversals giving rise to left–right confusions [63], and in part because learning-theory explanations came to be preferred to neurological ones. In a recent article, Dorothy Bishop reverses Orton's argument, suggesting that weak cerebral lateralization may itself result from impaired language learning [64]. Either way, the idea of an association between disability and failure of cerebral dominance may be due for revival, as recent studies have suggested that ambidexterity, or a lack of clear handedness or cerebral asymmetry, is indeed associated with stuttering [65] and deficits in academic skills [66], as well as mental health difficulties [67] and schizophrenia (see Box 1).

Although it may be the absence of asymmetry rather than its reversal that can be linked to problems of social or educational adjustment, left-handed individuals have often been regarded as deficient or contrarian, but this may be based more on prejudice than on the facts. Left-handers have excelled in all walks of life. They include five of the past seven US presidents, sports stars such as Rafael Nadal in tennis and Babe Ruth in baseball, and Renaissance man Leonardo da Vinci, perhaps the greatest genius of all time.
